# Association Between Helicobacter pylori Infection and Migraine: A Systematic Review

**DOI:** 10.7759/cureus.42747

**Published:** 2023-07-31

**Authors:** Sudiksha Sethia, Tayyaba Batool, Zarna Bambhroliya, Joel S Sandrugu, Michael Lowe, Oluwasemilore Okunlola, Shafaat Raza, Stephen Osasan, Pousette Hamid

**Affiliations:** 1 Research, California Institute of Behavioral Neurosciences & Psychology, Fairfield, USA; 2 Neurology, California Institute of Behavioral Neurosciences & Psychology, Fairfield, USA

**Keywords:** headache with aura, calcitonin gene-related peptide (cgrp), genetic variation, helicobacter pylori, migraine headaches

## Abstract

Migraine is a highly debilitating disease affecting humans worldwide. Despite having known this disease for a long time, not many studies have been done to search for a chronic infectious cause of migraine. The goal of this study was to look for an association between migraine and *Helicobacter pylori* infection. Following the Preferred Reporting Items for Systematic Review and Meta-Analyses (PRISMA) standards, we conducted the analysis and literature search using PubMed, Google Scholar and Cochrane databases. After applying the inclusion and exclusion criteria, the search technique produced a total of 10 articles including one cross-sectional study, two randomized controlled trials (RCTs), one cohort study, five case-control studies and one meta-analysis. Analysis of these studies revealed that there could be an association between *Helicobacter pylori *infection and migraine, especially in the Asian population. However, the mechanism by which the infection could possibly cause this extra-gastric disorder needs further research and analysis.

## Introduction and background

Migraine is a common familial, episodic brain disorder characterized by periodic, usually unilateral, pulsatile headache accompanied by nausea and/or vomiting and increased sensitivity to light, sound and/or movement that begins in childhood, adolescence or early adulthood [1]. Migraines that are of moderate to severe intensity are highly disabling. It affects about 12-15% of the general population in the world [2,3] and has an annual estimated burden of about $19.6 billion in the United States of America (USA) and more than €27 billion in the European continent [4,5].

Those suffering from migraine with aura (they make up about one-fourth to one-fifth [1] of the total migraine patients) (previously also known as classical migraine or neurological migraine) complain of visual disturbance or flashing white/multicolored lights (photopsia) or an enlarging blind spot with a shimmering edge (scintillating scotoma) or dazzling zigzag lights (called as fortification spectra or teichopsia) before the onset of headache. Other focal neurological symptoms like numbness, tingling or gait disturbance have also been described.

Earlier it was proposed that migraine is due to dilatation and constriction of cerebral arteries. This theory is no longer supported [6]. Although still unclear how an underlying genetic predisposition results in this complex brain disorder, an underlying genetic factor has been described as a risk factor for migraine headache. It is now theorized that a regional reduction in blood flow leading to cortical spreading depression or a wave of spreading oligemia could be responsible for the aura that precedes the headache [7]. Another hypothesis explaining the pathogenesis of migraine is the ‘neural mechanism’ where there is inflammation of fibers of the trigeminal nerve supplying the walls of the intra and extracranial vessels due to release of peptides like calcitonin gene-related peptide (CGRP) and substance P [8].

An underlying infectious cause that incites these events has not yet been conclusively found. The term “gastric headache” is not new. The link between chronic *Helicobacter pylori *infection and increased risk of migraine has been studied previously. This systematic review was undertaken to find out whether there is a causal association between *H. pylori* infection and migraine. This could help us reduce long-term morbidity associated with the disease and improve the quality of life of our patients to a considerable extent.

## Review

Methodology and results

The study selection, data extraction, and methodological quality assessment steps described below were adopted. 

Search Strategy

According to the Preferred Reporting Items for Systematic Reviews and Meta-analyses (PRISMA) guidelines [9], a systematic literature review was performed using databases starting from February 10, 2022. The last search was done on February 20, 2022. The databases included PubMed, Google Scholar and Cochrane.

For PubMed, the following Medical Subject Heading Terms (MeSH) and keywords were used: Helicobacter pylori OR H. pylori AND Migraine OR Migraine headache OR Migraine with aura OR Migraine without aura OR Unilateral Headache AND ("Helicobacter pylori/pathogenicity"[Majr]) OR "Helicobacter pylori/pathogenicity"[Mesh:NoExp] AND ("Migraine Disorders/microbiology"[Majr]) OR "Migraine Disorders/microbiology"[Mesh:NoExp]. The search only included original studies on human subjects published in the English language. Twenty-three relevant articles were obtained from PubMed.

For Google Scholar, the following keywords were used: Helicobacter pylori and migraine. Initial results obtained were 5530. To obtain more relevant articles, advanced search was applied where all the keywords, ‘Helicobacter pylori and Migraine’ were included in the title of the studies. Of the 28 results shown in Google Scholar, 20 were available. Further, six duplicates were found between PubMed and Google Scholar which were removed before screening.

For Cochrane, the following keywords were used, ‘Helicobacter pylori and Migraine’. Only two results were obtained. One of them was a duplicate result from PubMed, which was removed before starting the screening process.

Study Selection

The inclusion criteria included: Studies analyzing the association between patients diagnosed with migraine (by the International Headache Society Criteria) and *H. pylori* infection (detected by the rapid urease test or urea breath test or serum antibodies). Studies published only in English language were taken up for the systematic review. Studies analyzing possible pathophysiological/microbiological basis for migraine were also included. Cohort, case-control, cross-sectional study designs, or randomized controlled trials (RCTs) were included.

**Figure 1:**
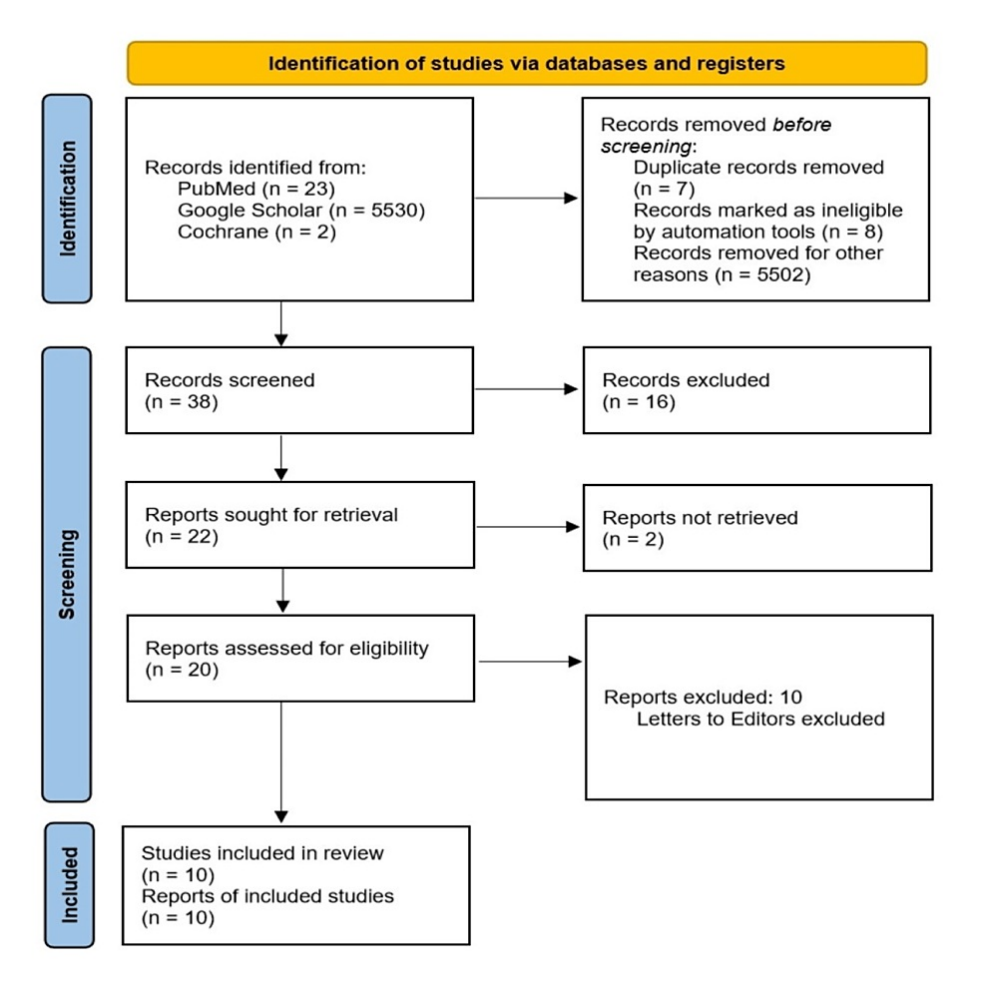
Preferred Reporting Items for Systematic Reviews and Meta-analyses (PRISMA) Flow Diagram of the Search Strategy

For final eligibility, full-text papers of abstracts that satisfied our inclusion criteria were evaluated. Studies published in other languages or those for which full text was not available were excluded. Case reports, case series, letters to editors and conference abstracts were excluded from the study. Figure 1 shows the PRISMA flow diagram of the search strategy used for the study.

Data Extraction

Data was extracted by two reviewers (S.S. and T.B.) independently using a standardized recording tool to document the study findings like the study design and setting, year of study, country where the study was done, number of participants in the study and their clinical characteristics, and the outcome of the study.

Methodological Quality Assessment 

Two investigators (S.S. and M.L.) independently evaluated the risk of bias of the included studies using the Newcastle Ottawa Quality Assessment Scale for case-control studies and cohort studies, the Cochrane risk of bias tool for an RCT, and the Assessment of Multiple Systematic Reviews (AMSTAR) Checklist for a meta-analysis.

The Newcastle Ottawa Quality Assessment Scale for case-control studies includes assessment on three domains: Selection, Comparability and Exposure with a maximum of nine stars for each study. The Newcastle Ottawa Quality Assessment Scale for cohort studies includes assessment on three domains: Selection, Comparability and Outcome with a maximum of nine stars for each study. The Cochrane risk of bias tool for the randomized clinical trial assesses the study for any risk of bias on the following six domains: Randomization, Intervention Assignment, Intervention Adherence, Missed Outcome Data, Measurement of Outcome, and Selection of Results. Accordingly, the overall risk of bias judgment for a study would be low risk of bias, some concerns or high risk of bias. The AMSTAR checklist consists of 16 questions with a maximum score of 16 for each study. To be called a high-quality study, a meta-analysis should score above 13 points. Table 1 below depicts the characteristics of all the included studies and Table 2 highlights the overall quality of the included studies.

**Table 1:** Characteristics of the Included Studies *Migraine Disability Assessment ** Cytotoxin Associated Gene A

AUTHORS OF THE STUDY	STUDY DESIGN	NAME OF THE JOURNAL	YEAR OF THE STUDY OR PUBLICATION YEAR	COUNTRY OF STUDY	NUMBER OF PARTICIPANTS	STUDY OUTCOME
Akbari et al [10].	Cross-sectional study	Iranian Journal of Neurology	May 2016 – May 2017	Iran	305	Of all patients with confirmed H. pylori infection, 69.5% had confirmed migraine; and of all patients without H. pylori infection only 3.27% had migraine. The study found a significant correlation between dyspepsia, migraine and H. pylori infection.
Ansari et al [11].	Case-control study	Iranian Journal of Neurology	2015	Iran	Cases – 84, Controls – 49	A significant difference was found in the levels of Immunoglobulin M antibody titres against H. pylori in migraine patients compared to controls implying the importance of active infection with H. pylori as a potential etiological factor in migraine. The study also found a significant positive correlation between Immunoglobulin G levels and severity of migraine in the patients.
Yiannopoulou et al [12].	Case-control study	The Journal of Headache and Pain	January 2003 – December 2003	Greece	Cases – 49, Controls – 51	In this study, 30 out of the 49 cases of migraine were found infected with H. pylori, while only 19 out of 51 controls were infected with H. pylori. The prevalence of H. pylori infection was significantly higher in patients with migraine with negative family history and no correlation to menstruation.
Faraji et al [13].	Randomized Controlled Trial	Pain Physician	2012	Iran	Treatment arm – 32, Control arm – 32	The reduction in MIDAS* score was significantly higher in treatment group when compared to control group. Patients with H. pylori were seen to have higher risk of migraine headache compared to those without infection. Eradication of H. pylori resulted in significant improvement of clinical symptoms of migraine.
Su et al [14].	Meta-analysis	World Journal of Gastroenterology	2014	-	Five case-control studies were included involving 903 patients.	The prevalence of H. pylori infection was significantly greater in migraine patients than in controls. Subgroup analysis found a significantly higher rate of infection with H. pylori in Asian patients with migraine, but no similar statistically significant higher infection rate in European patients with migraine. The odds ratios were 3.48 (95% Confidence Interval: 2.09 – 5.81) and 1.19 (C.I.: 0.86 – 1.65) respectively.
Ciancarelli et al [15].	Case-control study	Cephalalgia	2000	Italy	Cases – 30, Controls – 30	No significant increased levels of plasma oxidative by-products were found in migraineurs with H. pylori infection and migraineurs without H. pylori infection. Also, no significant difference was found in plasma nitric oxide metabolites between migraineurs and controls. This suggests that localized gastric release of H. pylori-related oxidative by-products may be inadequate to produce effects. No evident link seems to associate the oxidative states in migraineurs with H. pylori infection.
Pinessi et al [16].	Case-control study	Headache	2000	Italy	Cases – 103, Controls – 103	Chronic H. pylori infection is as present in patients with migraine as in controls and that this infection is not associated with any significant variation in the clinical features of the disease.
Gasbarrini et al [17].	Case-control study	Cephalalgia	2000	Italy	Cases – 175 (49 with aura, 126 without aura), Controls – 152	Prevalence of H. pylori infection was similar in migraine patients and in controls. The prevalence of Cag A** positive strains was slightly but not significantly greater in migraine patients. But, a significantly higher prevalence of Cag A positive strains was observed in patients affected by migraine with aura when compared with those without aura and in controls. The role of the highly virulent Cag A positive strain could be hypothesized in the pathogenesis of migraine with aura, however a double-blind cross-sectional eradication trial with a long-term follow-up remains mandatory to clarify the association.
Gasbarrini et al [18].	Cohort study	Hepatogastroenterology	October 1996 to January 1997	Italy	225	After eradication of H. pylori, migraine completely disappeared in 19 out of 81 eradicated patients. Intensity, duration and frequency of attacks of migraine significantly reduced in 77% of remaining eradicated patients. None of the non-eradicated subjects showed a significant reduction of clinical migraine.
Seyyed Majidi et al [19].	Randomized Controlled Trial	Caspian Journal of Neurological Sciences	October 2013 – November 2014	Iran	Treatment arm – 34, Control group – 46	The severity, duration and frequency of clinical headache attacks of all patients before, after six months, and after 12 months of treatment were analyzed. There was a significant decrease in severity, frequency and duration of migraine attacks at 12 months after H. pylori eradication in eradicated patients.

**Table 2:** Critical Appraisal/Quality Assessment of the Included Studies AMSTAR: Assessment of Multiple Systematic Reviews

STUDY	TYPE	QUALITY ASSESSMENT TOOL USED	CRITICAL APPRAISAL OF THE STUDY	OVERALL QUALITY OF THE STUDY
Akbari et al [10].	Cross-sectional study	Joanna Briggs Institute 2017, Critical Appraisal Checklist	Scores seven points out of eight	High quality
Ansari et al [11].	Case-control study	Newcastle Ottawa	Selection: 3* Comparability: 2* Exposure: 3*	High quality
Yiannopoulou et al [12].	Case-control study	Newcastle Ottawa	Selection: 3* Comparability: 1* Exposure: 2*	Some concerns
Faraji et al [13].	Randomized Controlled Trial	Cochrane risk of bias tool	Randomization: Some concerns Intervention Assignment: Low risk Intervention Adherence: Low risk Missed Outcome Data: Low risk Measurement of Outcome: Low risk Selection of Results: Low risk	Some concerns
Su et al [14].	Meta-analysis	AMSTAR Checklist	Scores 10 out of 16	Moderate quality
Ciancarelli et al [15].	Case-control study	Newcastle Ottawa	Selection: 4* Comparability: 1* Exposure: 3*	High quality
Pinessi et al [16].	Case-control study	Newcastle Ottawa	Selection: 4* Comparability: 2* (Both age and sex matched and also socio-economic status matched) Exposure: 3*	High quality
Gasbarrini et al [17].	Case-control study	Newcastle Ottawa	Selection: 4* Comparability: 2* (matched for age/sex/social background and geographical provenance) Exposure: 3*	High quality
Gasbarrini et al [18].	Cohort study	Newcastle Ottawa	Selection: 4* Comparability: 1* Outcome: 1*	Some concerns
Seyyedmajidi et al [19].	Randomized Controlled Trial	Cochrane risk of bias tool	Randomization: Low risk Intervention Assignment: High risk Intervention Adherence: High risk Missed Outcome data: Some concerns Measurement of Outcome: Some concerns Selection of results: Low risk	Low quality

Discussion

Headaches are classified into two categories: primary headache syndromes and secondary headaches. In primary headache syndromes the headache occurs without any exogenous cause. In secondary headache syndromes the headache is because of other pathological processes like head injury, meningitis, brain tumor, systemic infection, etc. Migraine is a primary headache syndrome as the pathology lies within. It has its origin at the intracranial vessels, duramater, trigeminal nerve and its ganglion, dorsal horns of C1 and C2 with modulatory centers in the diencephalon and brainstem [20].

Gastrointestinal Cause of Headache: Fact or Fallacy?

The association between gastric abnormalities and headache has long been speculated. The idea of “gastrointestinal cause of headache” was conceded even during the ancient times of Avicenna when traditional medicine was practised [21]. But the pathophysiological mechanism/s causing headache in a GI disorder is limited. Whether the gastric abnormalities are an effect of migraine or a cause of migraine is also debated. Studies done so far have found a positive correlation between patients suffering from dyspepsia and headaches [22]. This has been linked to the gut-brain axis which consists of the central nervous system, neuroendocrine system, neuroimmune system, hypothalamic-pituitary-adrenal (HPA) axis, autonomic nervous system, gut microbiota and the enteric nervous system. It has been found that gut microbiota influences the HPA axis to a significant level and there have been evidences of changes in mood, anxiety and other disorders with changes in the gut microbiome [23].

Pathogenesis of H. pylori: The Usual Microbiological Process

*H. pylori* lives in the gastric antral mucosa and causes a chronic infection persisting for years or sometimes decades and leading to gastritis and peptic ulcer disease (PUD) in most patients. In some patients the chronic infection can also lead to gastric adenocarcinoma and mucosa associated lymphoid tissue lymphomas (MALTomas). The production of protease, ammonia (by its urease enzyme) and other toxins by *H. pylori* accounts for the development of PUD.

Role of H. pylori in the Pathogenesis of Migraine: What Has Been Found Yet?

The 1990s: In the study done by Gasbarrini and colleagues in 1997, they found that around 23.45% of migraineurs suffering from *H. pylori* infection were completely relieved of future migraine attacks after the eradication of *H. pylori*. The remaining 76% of migraine patients with *H. pylori* infection stated significant reduction in frequency and severity of migraine attacks after the *H. pylori* eradication therapy [18].

However, it was also noted that not all patients with *H. pylori* infection suffered from migraine. And not all the migraine patients in the study were infected with *H. pylori.* Only 40% of the migraine patients tested positive for *H. pylori*. The theory put forth by Gasbarrini and colleagues was the multifactorial causation in the development of migraine [18]. Those with susceptible human leukocyte antigen (HLA) type and infected with some specific strains of *H. pylori* that released vasoactive mediators and cytokines were more likely to suffer from migraine and respond to *H. pylori* eradication therapy. Quality assessment of this study showed some concerns.

The 2000s: Ciancarelli and colleagues found that migraine patients had statistically significant elevated levels of plasma oxidative byproducts when compared with patients without migraine. In order to further study the role of *H. pylori *as a triggering factor for migraine in susceptible patients, they conducted a study to see whether *H. pylori *played a role in the raised levels of oxidative products (thiobarbituric acid reactive substances and nitric oxide metabolites) in migraine patients. They found no statistically significant elevated levels of oxidative byproducts in patients with *H. pylori *infection.

In another study done in the same year by Pinessi and colleagues, no significant difference in *H. pylori* positivity rate was found between migraineurs and non-migraineurs [16]. It is worthwhile to note that both these studies were high-quality studies.

A new insight came into the *H. pylori* and migraine conundrum when Gasbarrini and colleagues conducted another study, this time a high-quality study, and found that although the prevalence of *H. pylori* was similar in both migraine patients and controls, there was a significant increase in prevalence of the virulent Cag-A positive strains of *H. pylori* in migraine patients with aura compared to those without aura or in controls [17].

From 2010 to the present: In the studies that followed only a positive correlation was found between *H. pylori* and migraine. None of the studies could possibly explain a causal relationship between *H. pylori* and migraine or a pathological process explaining this positive association [10,11,13,19].

A meta-analysis done by Su and colleagues found that the prevalence of *H. pylori* was significantly increased in migraine patients when compared to controls. A subgroup analysis done by them revealed that the significant results were seen in Asian patients with migraine but not in European patients [14]. This again implicates a possible genetic predisposition to migraine that seems to be triggered by chronic infection with *H. pylori.*

Limitations and Strengths 

There have been some limitations while conducting this study. An important limitation was the scarcity of pertinent studies associated with the pathogenesis on this topic. Secondly, studies published on this topic in other languages were omitted from analysis. Thirdly, our study did not include animal studies and entirely looked at human research. Also, some studies with texts that were not available as open-access articles were also excluded.

A significant strength of our study was focusing on all kinds of articles from cross-sectional studies, case-control studies, cohort studies, RCTs to meta-analyses. Secondly, no restriction on age criteria of the patient population in the participating studies was applied.

## Conclusions

In order to establish a conclusive association between *H. pylori *and migraine we need randomized controlled trials and further research in the pathophysiology of migraine. The answer to whether *H. pylori *tests should be done on all diagnosed migraine patients still needs to be researched. Nonetheless, not all nausea and vomiting in migraine patients is because of migraine and eradication of *H. pylori* in detected patients can cause significant relief and improve the quality of life of our patients from the gastrointestinal standpoint.
